# Supplementary material for: Antibiotic Use before, during, and after Seeking Care for Acute Febrile Illness at a Hospital Outpatient Department: A Cross-Sectional Study from Rural India
Source: Antibiotics (Basel). 2022 Apr 25;11(5):574. doi: 10.3390/antibiotics11050574 (PMC9138085; doi:10.3390/antibiotics11050574)
Supplement: Supplementary file 1 [file antibiotics-11-00574-s001.zip › antibiotics-1685303-supplementary.pdf]

Supplementary File

Table S1. WHO ATC classification, AWaRe category, oral and parenteral Defined Daily Doses (DDDs).

				DDDs	
Antibiotic Chemical Subgroup	ATC code	Antibiotic Type	AWaRe Category	Oral g	Parenteral g
Intestinal antibiotics	A07AA11	Rifaximin	Watch	0.6	-
Tetracyclines	J01AA02	Doxycycline	Access	0.1	0.1
Penicillins with extended spectrum	J01CA04	Amoxicillin	Access	1.5	3
Combinations of penicillins*	J01CR02	Amoxicillin and clavulanic acid	Access	1.5	3
Second-generation cephalosporins	J01DC02	Cefuroxime	Watch	0.5	3
Third-generation cephalosporins	J01DD01	Cefotaxime	Watch	-	4
	J01DD02	Ceftazidime	Watch	-	4
	J01DD04	Ceftriaxone	Watch	-	2
	J01DD08	Cefixime	Watch	0.4	-
	J01DD13	Cefpodoxime	Watch	0.4	-
	J01DD64	Cefpodoxime proxetil and clavulanic acid	Not recommended	0.4	-
Monobactams	J01DF01	Aztreonam	Reserve	-	4
Macrolides	J01FA10	Azithromycin	Watch	-	0.5
Aminoglycosides	J01GB03	Gentamicin	Access	-	0.24
Fluoroquinolones	J01MA01	Ofloxacin	Watch	0.4	0.4
	J01MA02	Ciprofloxacin	Watch	1	0.8
	J01MA06	Norfloxacin	Watch	0.8	-
	J01MA12	Levofloxacin	Watch	0.5	0.5
Combinations of antibacterials	J01RA09	Ofloxacin and ornidazole	Not recommended	2 UD=2 tabs	-
	J01RA13	Norfloxacin and tinidazole	Unclassified	2 UD=2 tabs	-
	J01RAXX	Cefixime and azithromycin	Not recommended	2 UD=2 tabs	-
Glycopeptide antibacterials	J01XA01	Vancomycin	Watch	-	2
Imidazole/Nitroimidazole derivatives	J01XD01/P01AB01	Metronidazole	Access	2	1.5
Nitrofuran derivatives	J01XE01	Nitrofurantoin	Access	0.2	-

*including β-lactamase inhibitors

Supplementary File

Table S2. Distribution of patient reported antibiotic use before outpatient visit across age groups in years, according to the WHO AWaRe classification by encounters and percentage.

	<5 (n=17)		5-17 (n=8)		18-34 (n=36)		35-49 (n=13)		50-65 (n=9)		Total (n=83)	
AWaRe Category	n	%	n	%	n	%	n	%	n	%	n	%
Access	5.0	29.4%	4.0	50.0%	7.0	19.4%	3.0	23.1%	2.0	22.2%	21.0	25.3%
Watch	10.0	58.8%	4.0	50.0%	29.0	80.6%	10.0	76.9%	7.0	77.8%	60.0	72.3%
Reserve	-	-	-	-	-	-	-	-	-	-	-	-
Not recommended	2.0	11.8%	-	-	-	-	-	-	-	-	2.0	2.4%
Unclassified	-	-	-	-	-	-	-	-	-	-	-	-
Total	17.0		8.0		36.0		13.0		9.0		83.0	100.0%

Table S3. Distribution of patient reported antibiotic prescription during outpatient visit across age groups in years, according to the WHO AWaRe classification by encounters and percentage.

	<5 (n=38)		5-17 (n=64)		18-34 (n=134)		35-49 (n=49)		50-65 (n=28)		Total (n=313)	
AWaRe Category	n	%	n	%	n	%	n	%	n	%	n	%
Access	9.0	23.7%	27.0	42.2%	66.0	49.3%	25.0	51.0%	15.0	53.6%	142.0	45.4%
Watch	28.0	73.7%	37.0	57.8%	64.0	47.8%	24.0	49.0%	12.0	42.9%	165.0	52.7%
Reserve	-	-	-	-	1.0	0.7%	-	-	-	-	1.0	0.3%
Not recommended	1.0	2.6%	-	-	2.0	1.5%	-	-	1.0	3.6%	4.0	1.3%
Unclassified	-	-	-	-	1.0	0.7%	-	-	-	-	1.0	0.3%
Total	38.0		64.0		134.0		49.0		28.0		313.0	100.0%

Table S4. Distribution of patient reported antibiotic use after outpatient visit across age groups in years, according to the WHO AWaRe classification by encounters and percentage.

	<5 (n=11)		5-17 (n=27)		18-34 (n=36)		35-49 (n=13)		50-65 (n=2)		Total (n=89)	
AWaRe Category	n	(%)	n	%	n	%	n	%	n	%	n	%
Access	2.0	18.2%	13.0	48.1%	10.0	27.8%	4.0	30.8%	-	-	29.0	32.6%
Watch	9.0	81.8%	13.0	48.1%	25.0	69.4%	8.0	61.5%	2.0	100.0%	57.0	64.0%
Reserve	-	-	-	-	-	-	-	-	-	-	-	-
Not recommended	-	-	-	-	-	-	-	-	-	-	-	-
Unclassified	-	-	1.0	3.7%	1.0	2.8%	1.0	7.7%	-	-	3.0	3.4%
Total	11.0		27.0		36.0		13.0		2.0		89.0	100.0%

Supplementary File

Table S5. Distribution of patient reported antibiotic use before outpatient visit by age groups in years and WHO ATC class in Defined Daily Doses (DDD).

		<5 (n=17)		5-17 (n=8)		18-34 (n=36)		35-49 (n=13)		50-65 (n=9)		Total (n=83)	
Antibiotic Chemical Subgroup	Antibiotic Type	DDD	%	DDD	%	DDD	%	DDD	%	DDD	%	DDD	%
Intestinal antibiotics	Rifaximin	-	-	-	-	-	-	-	-	-	-	-	-
Tetracyclines	Doxycycline	-	-	-	-	18.0	13.6%	8.0	22.1%	2.0	8.2%	28.0	12.0%
Penicillins with extended spectrum	Amoxycillin	1.9	8.5%	-	-	5.0	3.8%	2.5	6.9%	2.0	8.2%	11.4	4.9%
Combinations of penicillins*	Amoxycillin and clavulanic acid	1.7	7.5%	7.2	35.9%	5.1	3.8%	0.8	2.3%	-	-	14.8	6.3%
Second-generation cephalosporins	Cefuroxime	-	-	-	-	1.6	1.2%	-	-	-	-	1.6	0.7%
Third-generation cephalosporin	Cefotaxime	-	-	-	-	-	-	0.5	1.4%	0.5	-	0.5	0.2%
	Ceftazidime	-	-	-	-	-	-	-	-	-	-	-	-
	Ceftriaxone	-	-	5.3	26.4%	-	-	-	-	-	-	5.3	2.2%
	Cefixime	0.5	2.2%	1.0	5.0%	27.0	20.4%	4.0	11.0%	3.0	12.4%	35.5	15.2%
	Cefpodoxime	3.3	14.4%	-	-	8.0	6.1%	-	-	-	-	11.3	4.8%
	Cefpodoxime proxetil and clavulanic acid	2.0	8.9%	-	-	-	-	-	-	-	-	2.0	0.9%
Monobactams	Aztreonam	-	-	-	-	-	-	-	-	-	-	-	-
Macrolides	Azithromycin	4.7	20.7%	2.5	12.6%	22.5	17.0%	3.3	9.2%	2.0	8.2%	35.0	15.0%
Aminoglycosides	Gentamicin	-	-	-	-	-	-	-	-	-	-	-	-
Fluoroquinolones	Ofloxacin	3.5	15.6%	-	-	18.0	13.6%	3.0	8.3%	3.0	12.4%	27.5	11.8%
	Ciprofloxacin	-	-	-	-	26.1	19.7%	14.0	38.7%	2.0	8.2%	42.1	18.0%
	Norfloxacin	-	-	-	-	-	-	-	-	6.0	24.7%	6.0	2.6%
	Levofloxacin	-	-	4.0	20.1%	-	-	-	-	4.0	16.5%	8.0	3.4%
Combinations of antibacterials	Ofloxacin and ornidazole	5.0	22.2%	-	-	-	-	-	-	-	-	5.0	2.1%
	Norfloxacin and tinidazole	-	-	-	-	-	-	-	-	-	-	-	-
	Cefixime and azithromycin	-	-	-	-	-	-	-	-	-	-	-	-
Glycopeptide antibacterials	Vancomycin	-	-	-	-	-	-	-	-	-	-	-	-
Imidazole/Nitroimidazole derivatives	Metronidazole	-	-	-	-	0.4	0.3%	-	-	-	-	0.4	0.2%
Nitrofuran derivatives	Nitrofurantoin	-	-	-	-	-	-	-	-	-	-	-	-
Total		22.5		19.9		131.6		36.2		24.5		234.2	100%

*including β-lactamase inhibitors

Supplementary File

Table S6. Distribution of antibiotic prescription at outpatient visit across age groups in years and WHO ATC class in Defined Daily Doses (DDD).

		<5 (n=38)		5-17 (n=64)		18-34 (n=134)		35-49 (n=49)		50-65 (n=28)		Total (n=313)	
Antibiotic Chemical Subgroup	Antibiotic Type	DDD	%	DDD	%	DDD	%	DDD	%	DDD	%	DDD	%
Intestinal antibiotics	Rifaximin	-	-	-	-	-	-	-	-	-	-	-	-
Tetracyclines	Doxycycline	-	-	-	-	202.0	26.9%	48.0	19.8%	26.0	21.3%	276.0	19.5%
Penicillins with extended spectrum	Amoxycillin	0.5	0.5%	-	-	-		-	-	-	-	0.5	0.0%
Combinations of penicillins*	Amoxycillin and clavulanic acid	7.8	7.7%	45.7	21.9%	157.3	20.9%	68.8	28.4%	35.3	28.9%	314.9	22.3%
Second-generation cephalosporins	Cefuroxime	-	-	-	-	2.4	0.3%	6.0	2.5%	-	-	8.4	0.6%
Third-generation cephalosporins	Cefotaxime	-	-	-	-	2.2	0.3%	1.8	0.7%	2.5	2.0%	6.5	0.5%
	Ceftazidime	-	-	-	-	-		0.4	0.2%	-	-	0.4	-
	Ceftriaxone	0.3	0.3%	-	-	5.0	0.7%	1.0	0.4%	-	-	6.3	0.4%
	Cefixime	20.4	20.2%	54.5	26.2%	57.5	7.7%	5.5	2.3%	7.5	6.1%	145.4	10.3%
	Cefpodoxime	1.3	1.2%	-	-	8.0	1.1%	8.1	3.4%	-	-	17.4	1.2%
	Cefpodoxime proxetil and clavulanic acid	-	-	-	-	7.5	1.0%	-	-	-	-	7.5	0.5%
Monobactams	Aztreonam	-	-	-	-	0.8	0.1%	-	-	-	-	0.8	-
Macrolides	Azithromycin	54.7	54.1%	100.0	48.0%	188.0	25.0%	43.4	17.9%	5.0	4.1%	391.1	27.7%
Aminoglycosides	Gentamicin	-	-	-	-	-		-	-	-	-	0.0	0.0%
Fluoroquinolones	Ofloxacin	5.5	5.4%	7.0	3.4%	6.0	0.8%	2.5	1.0%	-	-	21.0	1.5%
	Ciprofloxacin	-	-	-	-	37.0	4.9%	26.1	10.8%	8.6	7.0%	71.7	5.1%
	Norfloxacin	-	-	-	-	21.0	2.8%	9.0	3.7%	24.5	20.0%	54.5	3.9%
	Levofloxacin	3.0	3.0%	-	-	-		-	-	-	-	3.0	0.2%
Combinations of antibacterials	Ofloxacin and ornidazole	5.0	5.0%	-	-	-		-	-	5.0	4.1%	10.0	0.7%
	Norfloxacin and tinidazole	-	-	-	-	3.0	0.4%	-	-	-	-	3.0	0.2%
	Cefixime and azithromycin	-	-	-	-	5.0	0.7%	-	-	-	-	5.0	-
Glycopeptide antibacterials	Vancomycin	1.1	1.1%	-	-	-		-	-	-	-	1.1	0.1%
Imidazole/Nitroimidazole derivatives	Metronidazole	1.5	1.5%	1.2	0.6%	38.2	5.1%	18.8	7.8%	7.8	6.4%	67.5	4.8%
Nitrofuran derivatives	Nitrofurantoin	-	-	-	-	10.5	1.4%	3.0	1.2%	-	-	13.5	1.0%
Total		101.0		208.4		751.3		242.4		122.2		1425.3	100%

*including β-lactamase inhibitors

Table S7. Distribution of patient reported antibiotic use after outpatient visit across age groups in years and WHO ATC class in Defined Daily Doses (DDD).

		<5 (n=11)		5-17 (n=27)		18-34 (n=36)		35-49 (n=13)		50-65 (n=2)		Total (n=89)	
Antibiotic Chemical Subgroup	Antibiotic Type	DDD	%	DDD	%	DDD	%	DDD	%	DDD	%	DDD	%
Intestinal antibiotics	Rifaximin	-	-	-	-	6.7	3.2%	-	-	-	-	6.7	1.7%
Tetracyclines	Doxycycline	-	-	-	-	-	-	-	-	-	-	-	-
Penicillins with extended spectrum	Amoxycillin	-	-	1.0	1.5%	3.3	1.6%	-	-	-	-	4.3	1.1%
Combinations of penicillins*	Amoxycillin and clavulanic acid	1.5	4.2%	20.0	30.0%	22.5	10.8%	12.5	21.9%	-	-	56.5	14.3%
Second-generation cephalosporins	Cefuroxime	-	-	-	-	-	-	-	-	-	-	-	-
Third-generation cephalosporins	Cefotaxime	3.8	10.6%	-	-	3.8	1.8%	-	-	-	-	7.5	1.9%
	Ceftazidime	-	-	-	-	-	-	-	-	-	-	-	-
	Ceftriaxone	-	-	-	-	-	-	-	-	-	-	-	-
	Cefixime	-	-	17.5	26.2%	6.0	2.9%	6.0	10.5%	3.0	50.0%	32.5	8.2%
	Cefpodoxime	3.1	8.9%	-	-	-	-	-	-	-	-	3.1	0.8%
	Cefpodoxime proxetil and clavulanic acid	-	-	-	-	-	-	-	-	-	-	-	-
Monobactams	Aztreonam	-	-	-	-	-	-	-	-	-	-	-	-
Macrolides	Azithromycin	12.5	35.4%	25.8	38.7%	132.3	63.8%	25.0	43.9%	-	-	195.7	49.6%
Aminoglycosides	Gentamicin	3.4	9.7%	-	-	-	-	-	-	-	-	3.4	0.9%
Fluoroquinolones	Ofloxacin	5.0	14.2%	-	-	7.5	3.6%	-	-	-	-	12.5	3.2%
	Ciprofloxacin	-	-	2.4	3.6%	20.7	10.1%	3.0	5.3%	3.0	50.0%	29.1	7.4%
	Norfloxacin	-	-	-	-	4.5	2.2%	10.5	18.4%	-	-	15.0	3.8%
	Levofloxacin	9.8	17.0%	-	-	-	-	-	-	-	-	9.8	2.5%
Combinations of antibacterials	Ofloxacin and ornidazole	-	-	-	-	-	-	-	-	-	-	-	-
	Norfloxacin and tinidazole	-	-	4.0	-	4.0	-	3.0	-	-	-	11.0	2.8%
	Cefixime and azithromycin	-	-	-	-	-	-	-	-	-	-	-	-
Glycopeptide antibacterials	Vancomycin	-	-	-	-	-	-	-	-	-	-	-	-
Imidazole/Nitroimidazole derivatives	Metronidazole	-	-	0.6	-	3.0	-	3.8	-	-	-	7.4	1.9%
Nitrofuran derivatives	Nitrofurantoin	-	-	-	-	-	-	-	-	-	-	-	-
Total		39.1		71.4		214.3		63.8		6.0		394.5	100%

*including β-lactamase inhibitors

Supplementary File

Table S8. Distribution of antibiotic encounters for all 1000 patients by presumptive diagnosis and AWaRe category, in encounters and percentages.

	Presumptive diagnosis																	
	AVI (n=601)		UTI (n=117)		URTI (n=92)		LRTI (n=58)		Gastro. (n=58)		Typhoid (n=40)		Malaria (n=15)		Other* (n=19)		Total (n=1000)	
AWaRe Category	n	%	n	%	n	%	n	%	n	%	n	%	n	%	n	%	n	%
Access	50.0	8.3%	34.0	29.1%	22.0	23.9%	13.0	22.4%	10.0	17.2%	6.0	15.0%	5.0	33.3%	2.0	10.5%	143.5	14.3%
Watch	79.0	13.1%	28.0	23.9%	17.0	18.5%	13.0	22.4%	11.0	19.0%	10.0	25.0%	2.0	13.3%	5.0	26.3%	166.4	16.6%
Reserve	1.0	0.2%	-	-	-	-	-	-	-	-	-	-	-	-	-	-	1.0	-
Not recommended	2.0	0.3%	-	-	1.0	1.1%	-	-	1.0	1.7%	-	-	-	-	-	-	4.0	-
Unclassified	-	-	1.0	0.9%	-	-	-	-	-	-	-	-	-	-	-	-	1.0	-
Sub-total AWaRe	132.0	22.0%	63.0	53.8%	40.0	43.5%	26.0	44.8%	22.0	37.9%	16.0	40.0%	7.0	46.7%	7.0	36.8%	313.0	31.3%
No antibiotics	469	78.0%	54.0	46.2%	52.0	56.5%	32.0	55.2%	36.0	62.1%	24.0	41.4%	8.0	53.3%	12.0	63.2%	687.0	68.7%

AVI-Acute viral illness, UTI-urinary tract infection, URTI-Upper respiratory tract infection, LRTI-Lower respiratory tract infection, Gastro.-gastroenteritis, Other includes tuberculosis, appendicitis, severe acute malnutrition, rheumatic heart disease, abscess and septic arthritis. *including β-lactamase inhibitors

Supplementary File

Table S9. Distribution of antibiotics for all patients prescribed antibiotics at outpatient visit by presumptive diagnosis and ATC class in defined daily doses (DDDs).

		AVI (n=132)		UTI (n=63)		URTI (n=40)		LRTI (n=26)		Gastro. (n=22)		Typhoid (n=16)		Malaria (n=7)		Other (n=7)		Total (n=313)	
Antibiotic Chemical Subgroup	Antibiotic Type	DDD	%	DDD	%	DDD	%	DDD	%	DDD	%	DDD	%	DDD	%	DDD	%	DDD	%
Intestinal antibiotics	Rifaximin	-		-		-		-	-	-	-	-	-	-	-	-	-	0.0	0.0%
Tetracyclines	Doxycycline	112.0	17.4%	92.0	30.4%	20.0	12.2%	-	-	20.0	25.0%	24.0	26.8%	2.0	9.6%	6.0	15.3%	276.0	19.5%
Penicillins with extended spectrum	Amoxycillin	0.5	0.1%	-	-	-	-	-	-	-	-	-	-	-	-	-	-	0.5	0.0%
Combinations of penicillins*	Amoxycillin and clavulanic acid	118.5	18.5%	57.8	19.1%	63.7	38.9%	34.8	40.1%	13.7	17.1%	6.1	6.8%	11.7	55.8%	8.8	22.4%	314.9	22.3%
Second-generation cephalosporins	Cefuroxime	2.4	0.4%	-	-	-	-	-	-	-	-	6.0	6.7%	-	-	-	-	8.4	0.6%
Third-generation cephalosporins	Cefotaxime	2.7	0.4%	-	-	3.8	2.3%	-	-	-	-	-	-	-	-	-	-	6.4	0.5%
	Ceftazidime	0.4	0.1%	-	-	-	-	-	-	-	-	-	-	-	-	-	-	0.4	0.0%
	Ceftriaxone	3.0	0.5%	-	-	-	-	-	-	0.3	0.3%	-	-	-	-	3.0	7.7%	6.3	0.4%
	Cefixime	50.5	7.9%	40.5	13.4%	7.1	4.4%	5.5	6.4%	6.0	7.5%	18.8	20.9%	6.0	28.7%	11.0	28.1%	145.4	10.3%
	Cefpodoxime	12.4	1.9%	5.0	1.6%	-	-	-	-	-	-	-	-	-	-	-	-	17.4	1.2%
	Cefpodoxime proxetil and clavulanic	7.5	1.2%	-	-	-	-	-	-	-	-	-	-	-	-	-	-	7.5	0.5%
Monobactams	Aztreonam	0.8	0.1%	-	-	-	-	-	-	-	-	-	-	-	-	-	-	0.8	0.1%
Macrolides	Azithromycin	261.3	40.7%	8.3	2.7%	54.5	33.3%	39.2	45.2%	-	-	21.0	23.4%	-	-	6.7	17.1%	391.0	27.7%
Aminoglycosides	Gentamicin	-	-	-	-	-	-	-	-	-	-	-	-	-	-	-	-	0.0	0.0%
Fluoroquinolones	Ofloxacin	14.0	2.2%	-	-	2.5	1.5%	-	-	4.5	5.6%	-	-	-	-	-	-	21.0	1.5%
	Ciprofloxacin	40.0	6.2%	3.0	1.0%	1.2	0.7%	3.0	3.5%	19.5	24.4%	5.0	5.6%	-	-	-	-	71.7	5.1%
	Norfloxacin	-	-	54.5	18.0%	-	-	-	-	-	-	-	-	-	-	-	-	54.5	3.9%
	Levofloxacin	-	-	-	-	-	-	3.0	3.5%	-	-	-	-	-	-	-	-	3.0	0.2%
Combinations of antibacterials	Ofloxacin and ornidazole	5.0	0.8%	-	-	-	-	-	-	5.0	6.3%	-	-	-	-	-	-	10.1	0.7%
	Norfloxacin and tinidazole	-	-	3.0	1.0%	-	-	-	-	-	-	-	-	-	-	-	-	3.0	0.2%
	Cefixime and azithromycin	-	-	-	-	5.0	3.1%	-	-	-	-	-	-	-	-	-	-	5.0	0.4%
Glycopeptide antibacterials	Vancomycin	-	-	-	-	-	-	1.1	1.3%	-	-	-	-	-	-	-	-	1.1	0.1%
Imidazole/Nitroimidazole derivatives	Metronidazole	11.2	1.7%	25.5	8.4%	6.0	3.7%	-	-	11.0	13.8%	8.8	9.8%	1.2	5.7%	3.8	9.7%	67.5	4.8%
Nitrofuran derivatives	Nitrofurantoin	-	-	13.5	4.5%	-	-	-	-	-	-	-	-	-	-	-	-	13.5	1.0%
Total		642.1	45.0%	303.1	21.3%	163.7	11.5%	86.6	6.1%	79.9	5.6%	89.7	6.3%	20.9	1.5%	39.2	2.8%	1425.3	100.0%

*including β-lactamase inhibitors

AVI-Acute viral illness, UTI-urinary tract infection, URTI-Upper respiratory tract infection, LRTI-Lower respiratory tract infection, Gastro.-gastroenteritis, Other includes tuberculosis, appendicitis, severe acute malnutrition, rheumatic heart disease, abscess and septic arthritis.
